# 

**Published:** 1892-09

**Authors:** 


### CONTENTS—SEPTEMBER, 1892—VOL. VIII, NO. 3.

ORIGINAL ARTICLES—

A Case of Cystic Degeneration of both Ovaries, following a street car accident. Jas. Kennedy, M. D., San Antonio, Texas . . . . . . . 85
Retroversion of the Uterus. W. J. Matthews, Austin . . . . . . . . 91
Compound Fracture of Femoral Boue; Resection of Fractured Ends, Recovery. R. Menger, M. D., San Antonio . . . . . . . . . . . . 98

SELECTED ARTICLES—

Is there a Vegetable Pepsin . . . . . . . . . . . . . . . . . . . . 100

SOCIETY NOTES—

Joint Session Central Texas and Austin District Medical Societies . . 103

EDITORIAL—

Principle vs. Expediency . . . . . . . . . . . . . . . . . . . . . 105
Cholera . . . . . . . . . . . . . . . . . . . . . . . . . . . . . . 108
Dr. Barker and the Pauper Insane Again . . . . . . . . . . . . . . 110
A Solace for Solis . . . . . . . . . . . . . . . . . . . . . . . . 112
Dead Again! . . . . . . . . . . . . . . . . . . . . . . . . . . . . 114
Dr. P. W. Johns, Hot Springs . . . . . . . . . . . . . . . . . . . 115

BOOK NOTICES—

Principles and Practice of Bandaging . . . . . . . . . . . . . . . 115
Naphey's Therapeutics . . . . . . . . . . . . . . . . . . . . . . . 116
The Physician Himself; 10th edition . . . . . . . . . . . . . . . . 116

MEDICAL NEWS AND MISCELLANY—

Items of Interest to Doctors . . . . . . . . . . . . . . . . . . . 117

PUBLISHER'S NOTES . . . . . . . . . . . . . . . . . . . . . . . . . 119

